# Melatonin significantly influences seed germination and seedling growth of *Stevia rebaudiana* Bertoni

**DOI:** 10.7717/peerj.5009

**Published:** 2018-06-22

**Authors:** Magdalena Simlat, Agata Ptak, Edyta Skrzypek, Marzena Warchoł, Emilia Morańska, Ewa Piórkowska

**Affiliations:** 1 Department of Plant Breeding and Seed Science, University of Agriculture in Krakow, Krakow, Poland; 2 Department of Biotechnology, The Franciszek Górski Institute of Plant Physiology Polish Academy of Sciences, Krakow, Poland

**Keywords:** Antioxidant enzymes, Germination energy, Pigments, Germination capacity, Seedling development, Stevia

## Abstract

**Background:**

Melatonin (MEL) is a signaling molecule in plants that affects developmental processes during vegetative and reproductive growth. Investigations have proved that exogenously applied MEL also has the potential to improve seed germination and plant development.

**Methods:**

In the present study, seeds of stevia, a species with a very low germination rate, were germinated on an agar gel (AG) containing MEL at various concentrations (5, 20, 100, and 500 µM) in light. Seeds germinated on AG without MEL were used as controls. For the first 24 or 48 h of germination, the seeds were maintained in darkness as a pre-incubation step. Some seeds were not exposed to this pre-incubation step.

**Results:**

At concentrations of 20 and 5 µM, MEL significantly improved germination, but only in seeds pre-incubated in darkness for 24 h (*p* < 0.001). At concentrations of 100 and 500 µM, MEL had an inhibitory effect on germination, regardless of the pre-incubation time. Melatonin also affected plantlet properties. At a concentration of 20 µM, MEL increased plantlet fresh weight and leaf numbers. At a concentration of 5 µM, it promoted plantlet height. Regarding root development, the most favorable MEL concentration was 500 µM. Biochemical analysis revealed that MEL promoted higher pigment concentrations but hampered superoxide dismutase activity. On the other hand, the concentrations of sugars and phenolics, as well as the activities of catalase and peroxidase, increased at a MEL concentration of 500 µM.

**Discussion:**

The results suggest that MEL can improve germination of positively photoblastic stevia seeds and that it can play a role in plantlet development. However, the effects observed in the present study depended on the quantity of MEL that was applied.

## Introduction

Melatonin (MEL) (N-acetyl-5-methoxytryptamine), which was discovered in 1958 in the bovine pineal gland (Lerner et al., 1958), is one of the best studied biological molecules. In the early 1990s, MEL was detected in a unicellular photosynthetic organism, *Gonyaulax polyedra*. Since then, research concerning the role of MEL in plants has been ongoing (Dubbels et al., 1995; Hattori et al., 1995). To date, MEL has been detected in more than 300 plant species, including both monocotyledonous and dicotyledonous families (Manchester et al., 2000; Chen et al., 2003; Zohar et al., 2011; Arnao, 2014). Melatonin has been detected in various plant tissues, including roots, shoots, leaves, fruits, and seeds (Murch, Simmons & Saxena, 1997; Nawaz et al., 2016). The primary sites of MEL production are chloroplasts. Serotonin *N*-acetyltransferase, the enzyme responsible for the synthesis of MEL, has been identified in chloroplasts of rice plants (Byeon et al., 2014) and chloroplasts of red algae (Byeon et al., 2015). Although the role of MEL in animals is well known, its possible role in plants is less clear. Various studies have suggested that MEL can act as a plant growth regulator (Murch & Saxena, 2002; Arnao & Hernandez-Ruiz, 2006) and biostimulator in stressful situations (Arnao & Hernandez-Ruiz, 2013; Posmyk et al., 2008; Tan et al., 2007; Wei et al., 2015). Pre-treatment with MEL has increased seed germination of *Brassica oleracea rubrum*, *Cucumis sativus*, and *Phacelia tanacetifolia* (Posmyk et al., 2008; Posmyk et al., 2009; Tiryaki & Keles, 2012). The use of MEL in seed pre-treatment has also affected the future growth of plants. Used for soybean seed priming, MEL increased leaf size, plant height, and seed number (Wei et al., 2015). In canary grass, wheat, barley, and oats, MEL stimulated stem elongation (Hernandez-Ruiz, Cano & Arnao, 2005), and it induced cotyledon elongation in lupin (Hernandez-Ruiz & Arnao, 2008). In addition, MEL stimulated rhizogenesis in lupin (Arnao & Hernandez-Ruiz, 2007), cucumber (Zhang et al., 2013), rice (Park & Back, 2013), and pomegranate (Sarrou, Therios & Dimassi-Theriou, 2014). Its stimulating effect on root growth in *Brassica juncea* has also been proven (Chen et al., 2009). Furthermore, MEL has played a role in regulating the growth of *in vitro* cultures. For example, in sweet cherry explants, endogenous MEL modulated plant morphogenesis, changing the rate of rhizogenesis and caulogenesis (Murch & Saxena, 2002; Murch, Campbell & Saxena, 2001; Sarropoulou, Therios & Dimassi-Theriou, 2012). The possible role of MEL in apical dominance or the branching response has also been studied (Wang et al., 2014). Studies showed that MEL slowed induced senescence, delaying total chlorophyll loss (Arnao & Hernandez-Ruiz, 2009; Wang et al., 2012; Zhang et al., 2016; Zhang et al., 2017). Some authors have pointed to possible effects of MEL on chlorophyll-degrading enzyme genes (Wang et al., 2013b; Weeda et al., 2014; Zhang et al., 2016). Under conditions of stress, MEL improved the photosynthetic efficiency of chloroplasts by preserving chlorophyll. Research also showed that MEL had antioxidant properties, protecting plants against abiotic stress. Exogenous MEL enhanced reactive oxygen species (ROS) and the activities of scavenging enzymes (Rodriguez et al., 2004; Bałabusta, Szafrańska & Posmyk, 2016; Wang et al., 2016; Zhang et al., 2016; Wang, Reiter & Chan, 2018), such as catalase (CAT; EC 1.11.1.6), peroxidase (POD; EC 1.11.1.7), and superoxide dismutase (SOD; EC 1.15.1.1), which played a crucial role in decreasing and eliminating ROS.

Stevia is a perennial herb belonging to the Asteraceae family. It plays an important role in the human diet, mainly due to the presence of steviol glycosides. According to reports, steviol glycosides are about 200 times sweeter than sugar, yet much less calorific (Panpatil & Polasa, 2008; Madan et al., 2010; Yadav et al., 2011). In addition, steviol glycosides are resistant to high temperatures. Thus, they can be added to thermally processed food products. Owing to these properties, steviol glycosides are acceptable to both diabetic and phenyloketonuria patients (Guens, 2000). Stevia is naturally propagated by seeds. However, due to a low germination rate, propagation by seeds is not widely used in commercial stevia production. The reasons for low germinability are not well-defined; it could be related to environmental factors, such as low humidity or extreme temperature (Murdoch & Ellis, 2000). Moreover, as reported by Madan et al. (2010), excessive rainfall during pollination can affect both the yield and germination of stevia seeds. Alternatively, stevia may be propagated by stem cuttings or tissue culture, but these methods limit the large-scale production of plant material and are not cost effective (Yadav et al., 2011). There are some previous reports related to optimizing stevia seed germination. For example, Takahashi, Melges & Carneiro (1996) and Simlat et al. (2016) reported that the optimal temperature for stevia seed germination was 25 °C. Another report by Simlat et al. (2016) indicated that light had a positive influence on stevia seed germination. According to Abdullateef & Osman (2011), red light enhanced the germination potential of stevia seeds. In a study of the effect of light-emitting diodes (LEDs) on germination, Simlat et al. (2016) showed that blue LEDs had the most favorable effects on germination. Some studies of the use of phytohormones for improving stevia seed germination and different priming methods have also been published (Liopa-Tsakalidi et al., 2012; Hossa et al., 2017).

The positive effect of MEL on seed germination and plant growth, proven for several species, encouraged us to undertake the present research. In this study, we hypothesized that exogenous application of MEL to a germination substrate would enhance *S. rebaudiana* seed germination and improve subsequent developmental stages of obtained seedlings. Assuming this, in the present research, the percentage of seed germination (germination energy (GE) and germination capacity (GC); the plantlet morphology; the concentration of phenolics, soluble sugars, and pigments; as well as the activity of antioxidant enzymes were evaluated.

## Materials & Methods

### Plant material and germination experiment

The seeds of *S. rebaudiana* were kindly provided by POLAN Breeding and Seed Company, Poland. For the purpose of the experiment, the seeds were first surface sterilized according to Simlat et al. (2016). The sterilized seeds were placed directly on Petri dishes containing agar gel (AG) (dH_2_O solidified with 0.7% Difco Bacto Agar) supplemented with various concentrations of MEL (M5250; Sigma-Aldrich, St. Louis, MO, USA): 0 (control), 5, 20, 100, and 500 µM. To obtain the final concentrations, 10 mM MEL stock solution (23.22 mg of MEL was first dissolved in 1 ml of absolute (99.98%) EtOH and then supplemented with the appropriate volume of dH_2_O) was filter-sterilized (Millex –GP, 0.22 µm, Millipore, Burlington, MA, USA) and added, after autoclaving, to AG. The seeds were germinated in controlled plant growth chamber (Adaptis-A1000TC, Conviron, Winnipeg, Canada) at 25 °C under white fluorescent light with intensity expressed as photosynthetic photon flux density (PPFD) of 60 µmol m^−2^s^−1^ (which we previously tested: Simlat et al., 2016) for 16 h/day at 70 ± 5% relative humidity. The seeds were directly germinated in light (0 h of pre-incubation) or were kept in darkness for 24 h and 48 h, before germination in light. The experiments were performed in four replications, each consisting of 25 seeds. The number of germinated seeds was recorded every day, starting from the first day after the seeds had been placed on Petri dishes. After seven and 21 days of incubation, GE (%) and GC (%) were determined, respectively.

### Scanning electron microscopy (SEM)

To observe the germination, non-pre-incubated seeds germinated on AG without MEL were chosen. After 0, 12, 24, and 48-hours of germination, embryos (without a seed coat) and seedlings were fixed with 2.5% (w/v) glutaraldehyde in 0.1 M phosphate buffer at pH 7.2–7.4 for 15 min. They were further dehydrated using a graded series of ethanol (30–100% v/v) and acetone (100%) and dried with liquid CO_2_ in a critical point dryer (Type E3100 Industrial, LADD, Wiliston, VT, USA). Samples were then coated with gold using a sputter coater (Jeol JFC-1100E, Tokyo, Japan) and, finally, were observed using a scanning electron microscope (Jeol model JSM 5410, Tokyo, Japan) (Pathan, Bond & Gaskin, 2008).

### Seedling growth conditions

Seedling growth and the development of plantlets took place on MS (Murashige & Skoog, 1962) medium without MEL. The medium was adjusted to pH 5.8 before autoclaving and supplemented with 3% sucrose and 0.7% agar. Ten well-developed seedlings with cotyledons, obtained from seeds germinated on AG enriched with each MEL concentration: 0 (control), 5, 20, 100, and 500 µM, and pre-incubated for 24 h in darkness, were placed on MS medium and incubated at 25 °C for a further four weeks. The light conditions were as follows: fluorescent light with intensity expressed as a PPFD of 120 µmol m^−2^s^−1^ for 16 h/day, and 70 ± 5% relative humidity (Adaptis-A1000TC, Conviron, Winnipeg, Canada). After a defined time period, morphological observations of the plantlets and biochemical analyses (determination of pigments, soluble sugars, phenolic concentrations, and activity of antioxidant enzymes) were made.

### Morphological observations of plantlets

Morphological features were described for four-week-old plantlets. All 10 plantlets, representing each MEL concentration applied for seed germination, were taken. The fresh weight (FW) and length of stems and roots were measured. The number of leaves and roots was also calculated. Each measurement was expressed as a mean for 10 replicates (one plant means one replication).

### Determination of pigments

Chlorophylls and carotenoids were extracted from fresh tissue samples (100 µg) in 1 ml of 80% ethanol for 12 h. After centrifugation (8,800× g, 15 min), an aliquot of extract was added to 96-wells micro-plate wells and absorbance was measured spectrophotometrically at 470, 648, and 664 nm on a micro-plate reader (Synergy 2, Bio-Tek, Winooski, VT, USA). The concentrations of chlorophyll *a* (Chl *a*), chlorophyll *b* (Chl *b*), and total carotenoids (Car) were determined according to Lichtenthaler & Wellburn (1983) and expressed per 1 g of tissue FW.

### Determination of soluble sugars

Soluble sugars were extracted using 80% ethanol from 100 µg fresh tissue samples. The amounts of total sugars were estimated after centrifugation (8,800× g, 15 min) by the phenol-sulfuric method (Dubois et al., 1956). The supernatant was mixed with 5% phenol and 95% sulfuric acid. The absorbance (*λ* = 490 nm) of the samples was estimated spectrophotometrically on a micro-plate reader (Synergy 2, Bio-Tek, Winooski, VT, USA). The total soluble sugar content was calculated as micrograms of glucose per 1 g of tissue FW.

### Determination of phenolics

For phenolic estimation, 5 mg samples of fresh tissue were homogenized in 1 ml of 80% ethanol and centrifuged at 8,800× g for 15 min. Subsequently, 30 µl of supernatant was mixed with 250 µl of 20% Na_2_CO_3_ and 70 µl of Folin-Ciocalteau reagent (Singleton & Rossi Jr, 1965). The absorbance (*λ* = 760 nm) of the samples was estimated spectrophotometrically using a 96-wells micro-plate reader (Synergy 2, Bio-Tek, Winooski, VT, USA). The total phenolics content was calculated as micrograms of chlorogenic acid per 1 g of tissue FW.

### Antioxidant enzymes activity assay

Fresh plant tissue (100 µg) was homogenized at 4 °C with phosphate buffer (pH 7.8) containing 0.01 M EDTA and 0.5% BSA. The homogenate was centrifuged at 6,000× g for 15 min. The activity of CAT was measured spectrophotometrically (*λ* = 240 nm) by the method of Aebi (1984). The reaction mixture consisted of the supernatant and 0.05 M phosphate buffer (pH 7.0) containing 0.1 mM EDTA and 0.03 M  H_2_O_2_. The activity of POD was measured by the method of Lück (1962). The measurement was carried out spectrophotometrically (*λ* = 485 nm) by measuring the amount of the products in 1% p-phenylenediamine (pPD) and the supernatant, in 0.05 M phosphate buffer containing 0.1 mM EDTA, pH 7.0. The reaction was started in the presence of 0.05 ml of 0.03 M H_2_O_2_. The activity of SOD was measured spectrophotometrically (*λ* = 595 nm) by the method of McCord & Fridovich (1969). One unit was defined as the amount of enzyme necessary for 50% inhibition of cytochrome *c* in a coupled system with xanthine and xanthine oxidase. The reaction kinetics for all enzymes was examined 60 s after initiation of the reaction.

### Data analysis

Differences in the percentage of germinating seeds at the seventh (GE) and 21st (GC) days of incubation were analyzed using a two-factorial analysis of variance (ANOVA) with MEL concentrations (0, 5, 20, 100, and 500 µM) and pre-incubation time (0, 24, and 48 h) as the factors. Seedling weight, stem and root length, number of leaves and roots, pigments, soluble sugars, phenolic concentrations, and antioxidant enzyme activity were also analyzed by one-way ANOVA. For the comparison of means, Duncan’s multiple test at the significance level of *p* < 0.05 implemented in the statistical package STATISTICA (data analysis software system), version 10.0 (http://www.statsoft.com) was used. The data are reported as means ± standard error (SE).

## Results

### Seed germination

**Figure 1:**
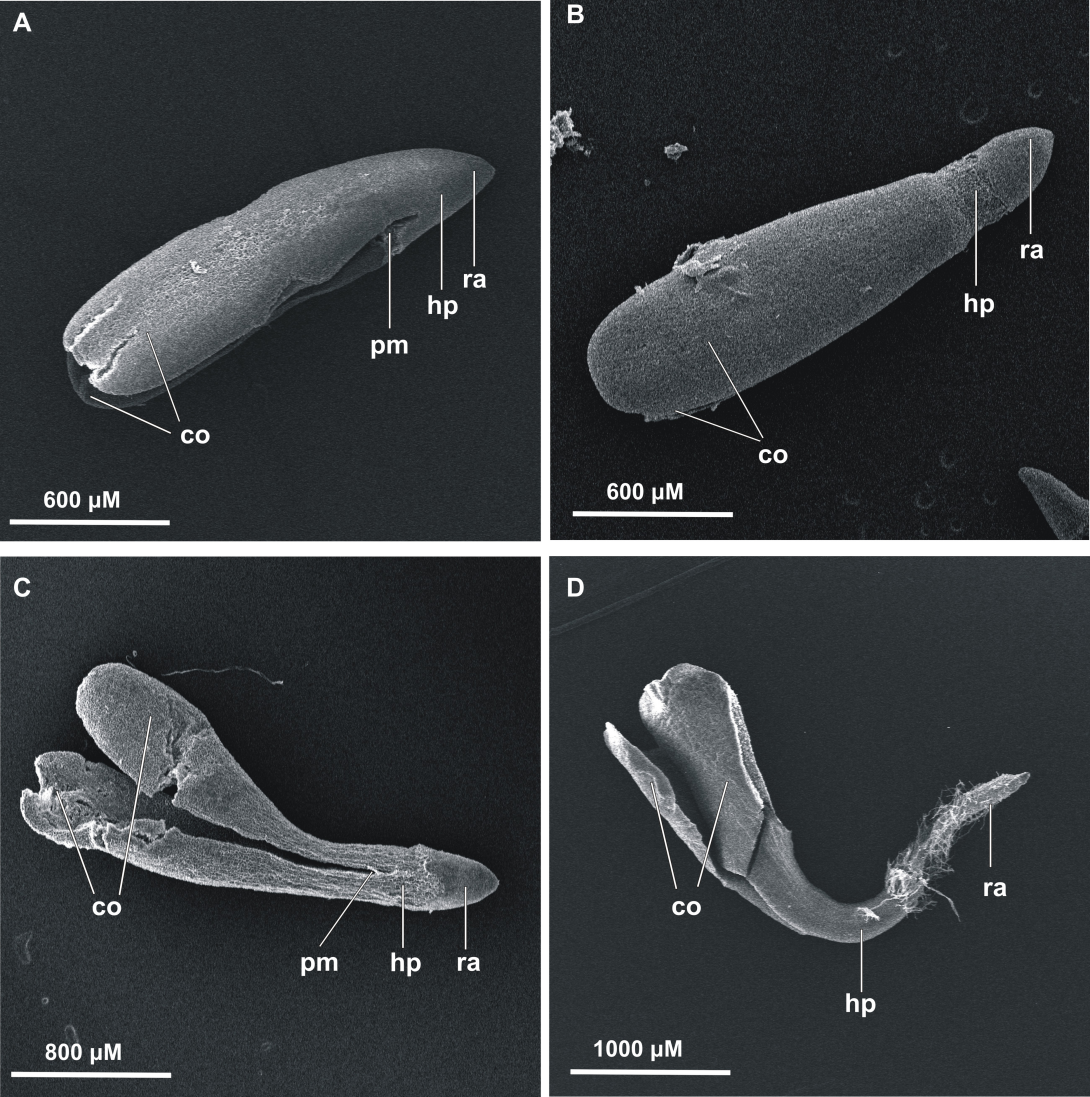
Scanning electron microscopy of germinated stevia seeds. (A) 0 h, (B) 12 h, (C) 24 h and (D) 48 h of incubation on AG (co, cotyledons; pm, plumule; hp, hypocotyl; ra, radicle).

**Figure 2:**
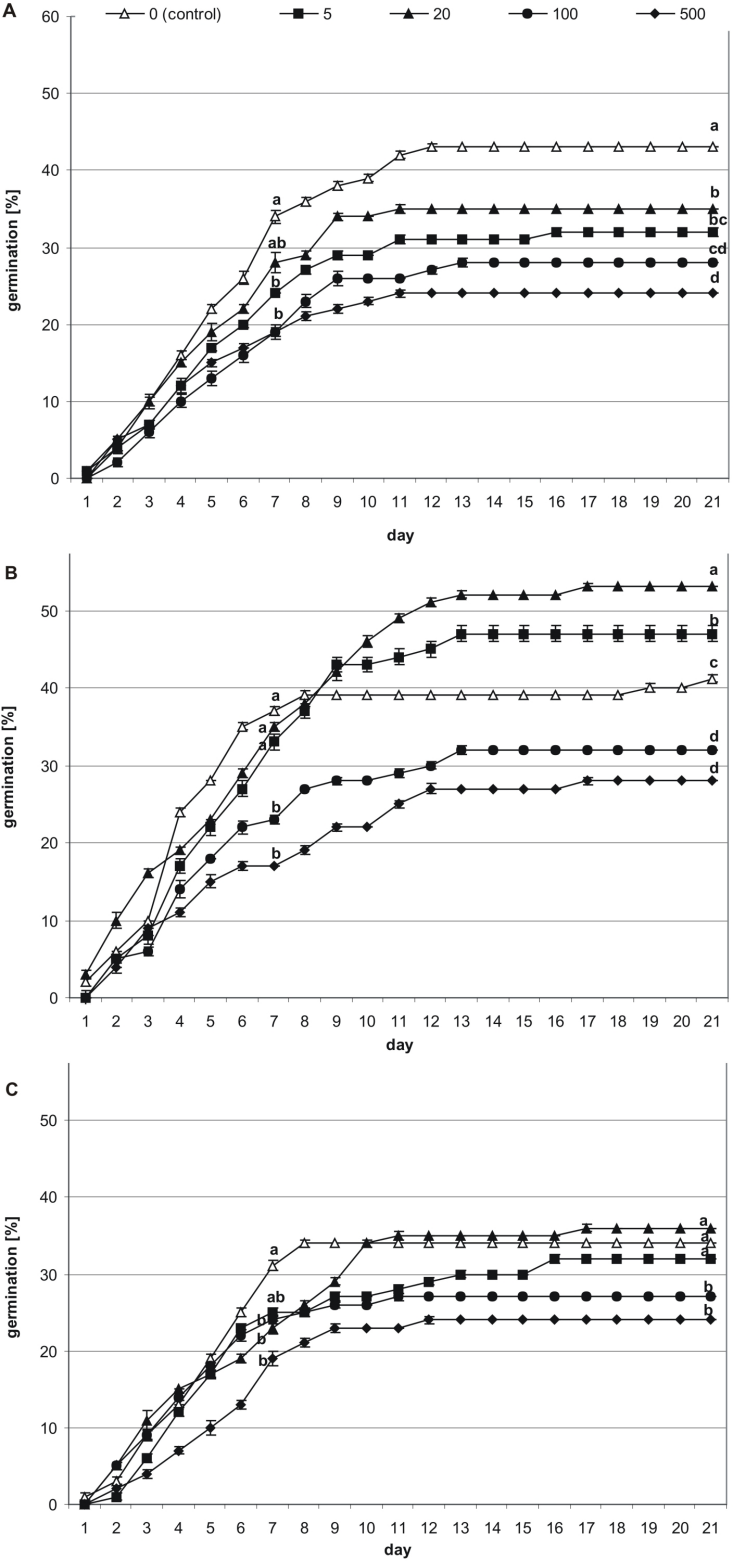
Effects of melatonin on stevia seed germination. The seeds were germinated on AG supplemented with various concentration of MEL (0, 5, 20, 100 and 500 µM) under different pre-incubation times: 0 h (A), 24 h (B) and 48 h (C). The results are the means of four replicates (*n* = 4) ±SE. Different letters indicate a significant difference in the germination at seventh (GE) and at the 21st (GC) days of incubation, at *p* < 0.05 according to ANOVA and Duncan’s test.

The first germinated seeds—those with green cotyledons at least protruding from the seed coat—were observed at 48 h of incubation. To present the germination process from imbibition to the formation of seedlings, stevia seeds without achenes were used (Fig. 1). However, the greatest increase in the number of germinated seeds was noted during the first nine days, irrespective of the substrate used and the initial pre-incubation step (Fig. 2). Stevia seeds have the potential to germinate even after a longer incubation period on a substrate, which our previous experiments (Simlat et al., 2016) have shown, and that is why the observations were carried out for 21 days.

Among seeds not pre-incubated in darkness (0 h) (Fig. 2A), the highest germination (GE = 34%, GC = 43%) was observed for control seeds. Considering the tested MEL concentrations, 20 µM had the most favorable effect on germination (GE = 28%, GC = 35%), although this was slightly lower than that of the control. The percentage of seed germination decreased in accordance with further increments in the MEL concentration in AG, and at MEL concentrations of 100 or 500 µM GE was at 19%, whilst at the final observation, GC reached 28% and 24%, respectively.

After 24 h of pre-incubation in darkness (Fig. 2B), 20 µM MEL had the most favorable effect on germination, with seed GC at 53%. This favorable effect was observed from the 10th day of incubation. Slightly fewer seeds germinated on AG with 5 µM MEL (GC = 47%), whereas GC in the control (AG without MEL) was 41%. The remaining MEL concentrations decreased seed germination, with the most negative effects observed when 500 µM MEL was added to the AG. Nevertheless, there were no significant differences in the germination percentages at MEL concentrations of 100 and 500 µM both for the seventh (GE = 23 and 17%, respectively) and 21st (GC = 32 and 28%, respectively) day of observation.

After 48 h of pre-incubation in darkness (Fig. 2C), the differences in the GE between tested MEL concentrations were not significant, and in all cases, the calculated percentages were lower compared to the control (AG without MEL). In regard to GC, the differences between the control, AG supplemented with 5 µM MEL, and AG supplemented with 20 µM MEL, were not significant. However, the GC observed for 20 µM MEL was higher compared to the control (36 and 34%, respectively). On the other hand, higher concentrations of MEL (100 and 500 µM) significantly inhibited stevia seed germination compared to the control (GC = 27 and 24%, respectively).

The addition of MEL to the AG significantly (*p* < 0.001) influenced stevia seed germination, which was especially evident after the analysis of seed GC (**Table 1**). It was observed that the lowest concentrations of MEL (5 and 20 µM) were more favorable for seed GC than the highest concentrations (100 and 500 µM MEL). In addition, pre-incubation time in darkness had a significant (*p* < 0.001) effect on seed GC. As evident for control seeds (germinated on AG without MEL), the highest GC was observed for seeds not pre-incubated in darkness (43%), and the lowest was observed for seeds pre-incubated for 48 h (34%). The interaction between MEL concentration and pre-incubation time was also significant (*p* < 0.001). The addition of MEL to the AG at concentrations of 5 µM or 20 µM eliminated the negative influence of 24 h of darkness, with the observed percentage of germinated seeds (GC) significantly higher, even when compared with that of control seeds germinated without pre-incubation. Due to the observed positive effect of MEL on seed GC after 24 h of pre-incubation (*p* < 0.001), the seedlings obtained in that experiment were transferred to MS medium for further growth.

**Table 1:** Results of variance analysis applied to the percentage of germination at seventh (GE) and 21 st (GC) days of incubation, morphological observations, and biochemical analysis.

	Source	*df*	MS	*F*-value	*p*
GE (%)	MEL concentration (M)	4	442.93	17.08^***^	0.00
	Pre-incubation time (T)	2	129.87	4.99^*^	0.01
	*M* × *T*	8	45.53	1.75^ns^	0.11
	Error	45	26.04		
GC (%)	M	4	571.60	42.87^***^	0.00
	T	2	520.80	39.06^***^	0.00
	*M* × *T*	8	81.80	6.13^***^	0.00
	Error	45	13.33		
Fresh weight [g]	M	4	0.18	37.00^***^	0.00
	Error	45	0.01		
Shoot fresh weight [g]	M	4	0.07	29.18^***^	0.00
	Error	45	0.00		
Stem length (cm)	M	4	8.48	153.84^***^	0.00
	Error	45	0.06		
Internode length (cm)	M	4	0.37	32.91^***^	0.00
	Error	45	0.01		
Leaves/plantlets number	M	4	8.88	3.69^*^	0.01
	Error	45	2.41		
Roots number	M	4	79.15	52.84^***^	0.00
	Error	45	1.50		
Root length (cm)	M	4	0.56	17.42^***^	0.00
	Error	45	0.03		
Chl *a*	M	4	876.98	603.14^***^	0.00
	Error	10	1.45		
Chl *b*	M	4	263.66	119.72^***^	0.00
	Error	10	2.20		
Car	M	4	11.67	36.29^***^	0.00
	Error	10	0.32		
Soluble sugars	M	4	4,653.30	79.44^***^	0.00
	Error	10	58.60		
Phenolics	M	4	6.94	220.43^***^	0.00
	Error	10	0.03		
CAT	M	4	9,613.70	65.19^***^	0.00
	Error	10	147.50		
POD	M	4	248,630.00	356.89^***^	0.00
	Error	10	697.00		
SOD	M	4	0.08	142.41^***^	0.00
	Error	10	0.00		


**Notes.**

*** Significant at the 0.001 probability level.

* Significant at the 0.05 probability level.

ns Not significant.

### Plantlet morphology

**Figure 3:**
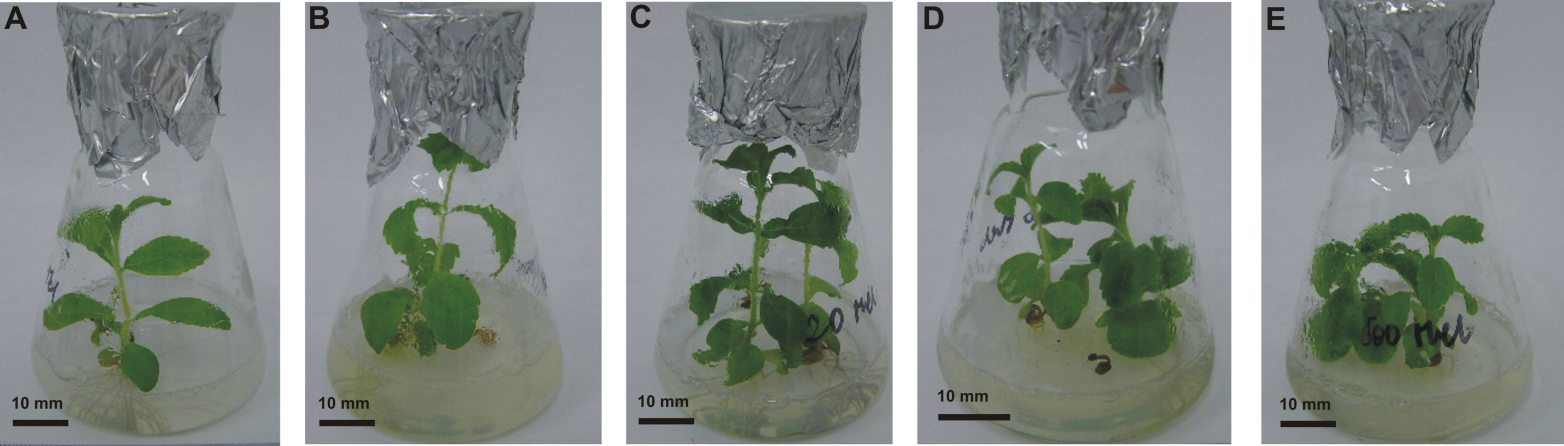
Effects of MEL on stevia shoots’ development. Plantlets obtained from seeds pre-incubated in darkness for 24 h, and germinated on AG supplemented with various concentration of MEL: 0 (A), 5 (B), 20 (C), 100 (D) and 500 µM (E), were grown for four weeks in *in vitro* conditions on MS medium. Photo credit: Magdalena Simlat.

**Figure 4:**
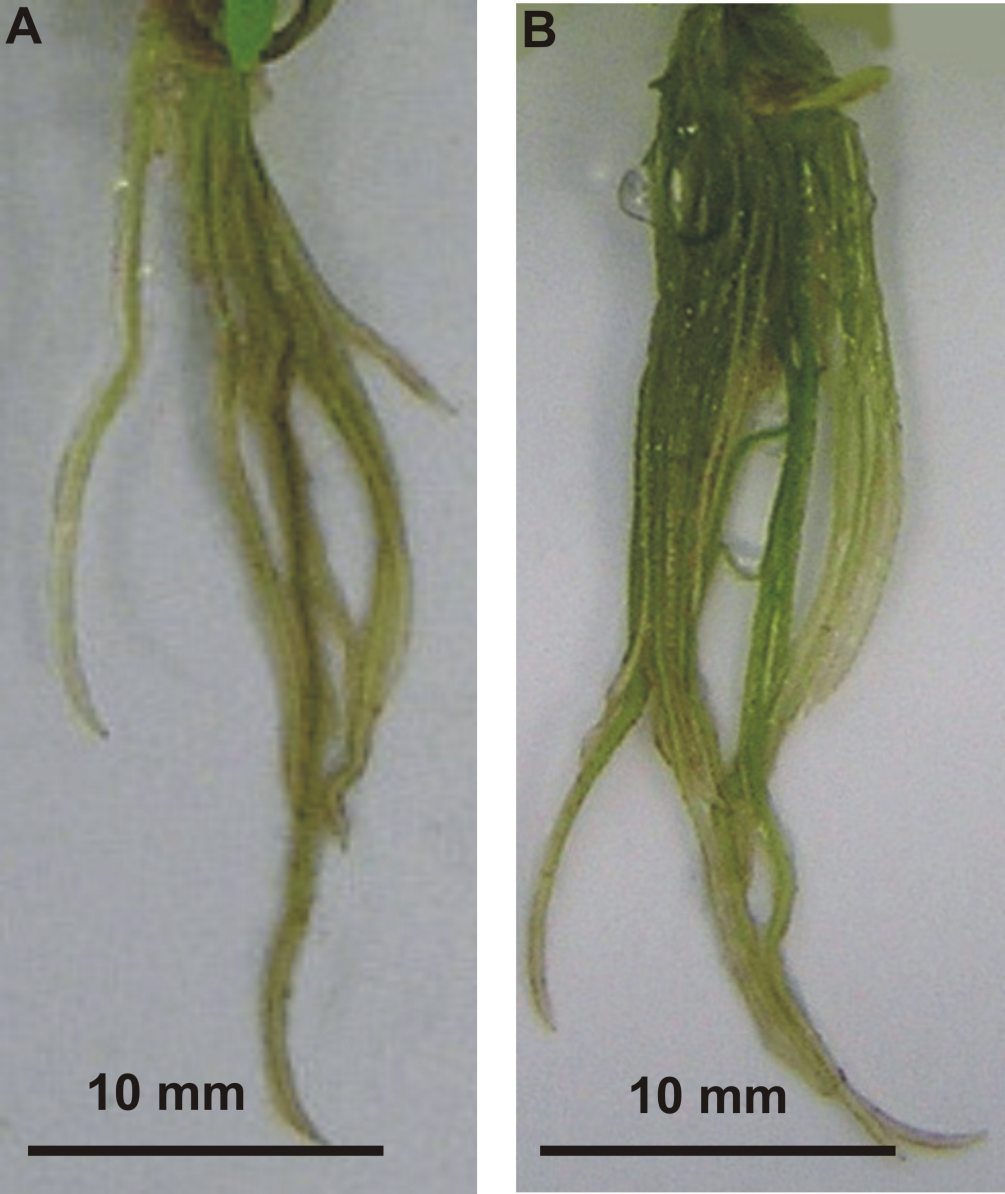
Effects of MEL on stevia roots’ development. Roots of the plantlets obtained from seeds pre-incubated in darkness for 24 h and germinated on AG without MEL (A) and on AG supplemented with 500 µM of MEL (B) were grown for four weeks in *in vitro* conditions on MS medium. Photo credit: Magdalena Simlat.

After four weeks of stevia seedling growth on MS medium without growth regulators, the morphological parameters of the obtained plantlets were assessed to observe the effect of MEL applied at the germination stage on the further growth of seedlings and the development of plantlets (**Tables 1 and **2**). Plantlets obtained from seeds germinated on 20 µM MEL had significantly (*p* < 0.001) higher total and aboveground FW (0.47 and 0.30 g, respectively). The other MEL concentrations significantly decreased the FW of stevia. On average, the total FW was 1.9 times lower than that of the control (0.33 g), whereas the aboveground FW was 1.5 times lower than that of the control (0.18 g). Melatonin at a concentration of 20 µM had the best stimulating effect on leaf development, with 11.6 leaves per plant, as compared with 9.8 leaves per plant in the control (*p* < 0.05). There was also a significant (*p* < 0.001) interaction between the MEL concentration and stem length. Seeds germinated on MEL at a concentration of 5 µM produced the longest plantlets, although significantly longer plantlets were also obtained with 20 µM MEL, as compared with the control. At higher MEL concentrations, the stem length decreased and seeds germinated on AG with the highest MEL concentration (500 µM) produced plantlets that were half as high as those obtained from seeds germinated on AG with the lowest tested concentration (5 µM). Similar outcomes were observed for internode lengths (**Table 2**, Fig. 3). In regard to the root system, germination on 500 µM MEL significantly (*p* < 0.001) enhanced (1.5-fold) the number of roots of obtained plantlets as compared with control plantlets (Fig. 4). The effects of the other MEL concentrations varied. However, in all cases, the root number was reduced compared with that of the control. On the other hand, MEL inhibited root elongation, as the longest roots (3.66 cm) developed in control plantlets. This inhibition effect increased in accordance with increments in the MEL concentration, and a concentration of 500 µM had the greatest inhibitory effect on root elongation (3.03 cm) (**Table 2**).

**Table 2:** Effects of melatonin on stevia growth in *in vitro* conditions. Melatonin at concentrations of 0 (control), 5, 20, 100 and 500 µM were used at the germination stage and obtained seedlings were grown for four weeks on MS medium. The means (*n* = 10) ± SE followed by different letters, are significantly different based on ANOVA followed by Duncan’s stest at *p* < 0.05.

MEL concentration (µM)	Fresh weight (g)	Shoot fresh weight (g)	Stem length (cm)	Internode length (cm)	Leaves/plantlets number	Roots number	Root length (cm)
0 (control)	0.33 ± 0.07 b	0.18 ± 0.05 b	3.64 ± 0.19 c	0.67 ± 0.19 b	9.8 ± 1.9 b	8.8 ± 1.4 b	3.66 ± 0.22 a
5	0.16 ± 0.07 c	0.12 ± 0.04 c	4.41 ± 0.28 a	0.83 ± 0.09 a	9.6 ± 1.4 b	6.7 ± 1.2 c	3.44 ± 0.14 b
20	0.47 ± 0.08 a	0.30 ± 0.08 a	3.87 ± 0.20 b	0.68 ± 0.14 b	11.6 ± 1.2 a	8.3 ± 0.9 b	3.39 ± 0.08 b
100	0.21 ± 0.07 c	0.12 ± 0.02 c	2.58 ± 0.22 d	0.46 ± 0.07 c	9.2 ± 1.4 b	6.0 ± 0.9 c	3.22 ± 0.27 c
500	0.15 ± 0.02 c	0.10 ± 0.02 c	2.21 ± 0.27 e	0.34 ± 0.05 d	9.6 ± 1.6 b	13.2 ± 0.1 a	3.03 ± 0.09 d

### Pigments

**Figure 5:**
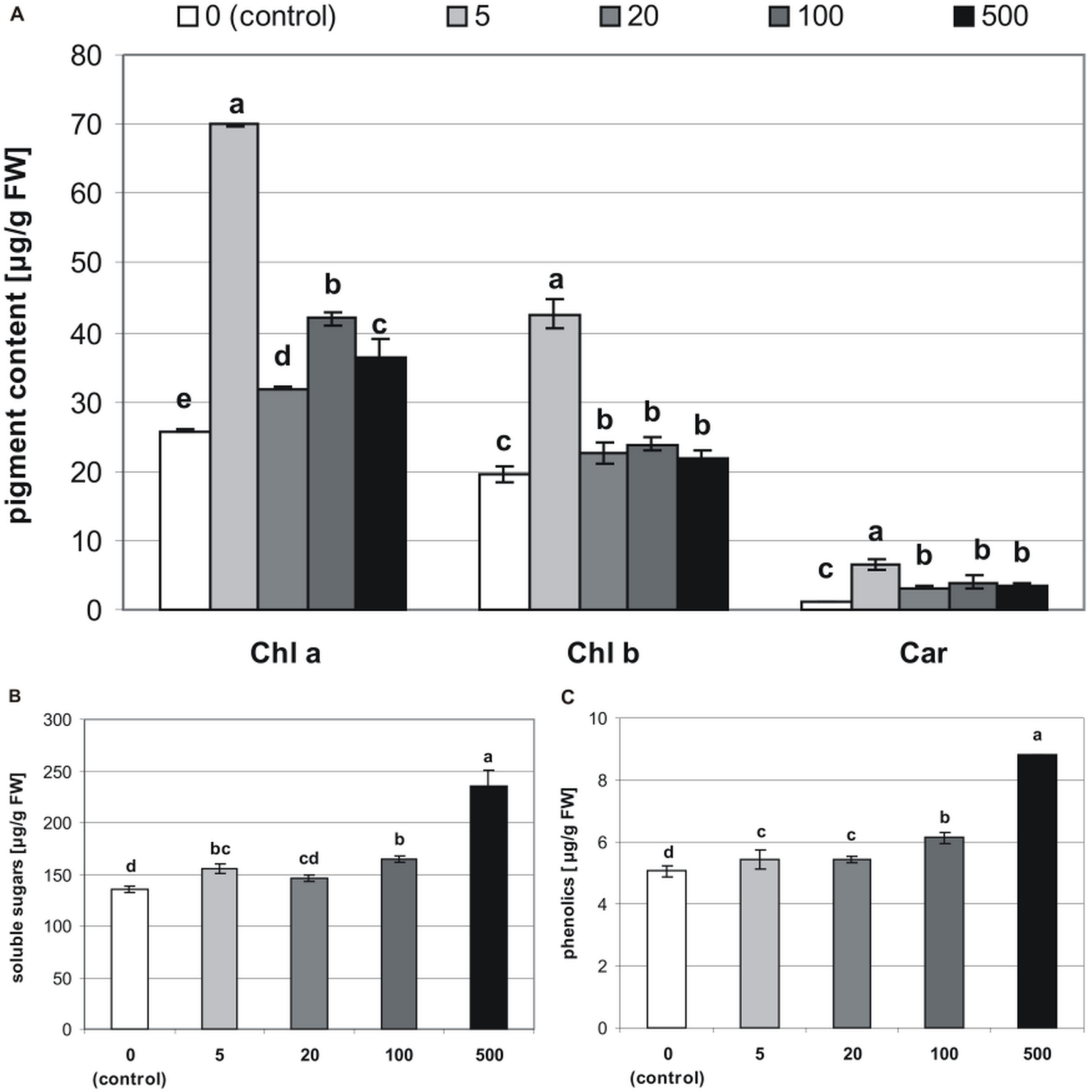
Effects of MEL on the content of pigments (A), soluble sugars (B) and phenolics (C) in four-week-old plantlets. Melatonin was used at the germination stage at concentrations of 0, 5, 20, 100 and 500 µM, and the seeds were pre-incubated in darkness for 24 h. The results are the means of three replicates (*n* = 3) ± SE. Different letters indicate a significant difference at *p* < 0.05 according to ANOVA and Duncan’s test.

The addition of MEL also affected concentrations of chlorophylls and carotenoids in stevia plantlets (**Table 1**). The highest concentration of Chl *a* (69.86 µg/g of FW) was found in plantlets obtained from seeds germinated on 5 µM MEL (Fig. 5A). The remaining MEL concentrations increased the concentration of Chl *a* as compared with that of the control (25.77 µg/g of FW). However, the values in all the other MEL treatments were about 50% lower than those detected in plantlets obtained from seeds germinated on 5 µM MEL. The profile of the Chl *b* content was similar to that of Chl *a*, although Chl *b* values in all the samples were lower than those of Chl *a*. The highest Chl *b* values (42.66 µg/g of FW) were detected in plantlets obtained from seeds germinated on 5 µM MEL. Regarding the levels of total carotenoids, the addition of 5 µM MEL at the germination stage had the most beneficial effects (6.62 µg/g of FW) in stevia plantlets. At the other concentrations of MEL, the levels of carotenoids were similar but higher (average 3.2 times) than those of control plantlets.

### Sugars

The tested MEL concentrations applied at the germination stage had a positive effect on soluble sugar levels in stevia plantlets (Table 1). These levels ranged from 136.1 to 235.4 µg/g of FW, with the lowest value detected in control plantlets and the highest value observed in plantlets obtained from seeds germinated on 500 µM MEL (Fig. 5B).

### Phenolics

Significantly (*p* < 0.001, Table 1) lower values of phenolics were detected in control plantlets (5.1 µg/g of FW), and the highest values were found in plantlets obtained from seeds germinated on 500 µM MEL. There were also significant differences between the phenolic levels detected in plantlets obtained from seeds germinated on other MEL concentrations (Fig. 5C).

### Antioxidant enzyme activity

**Figure 6:**
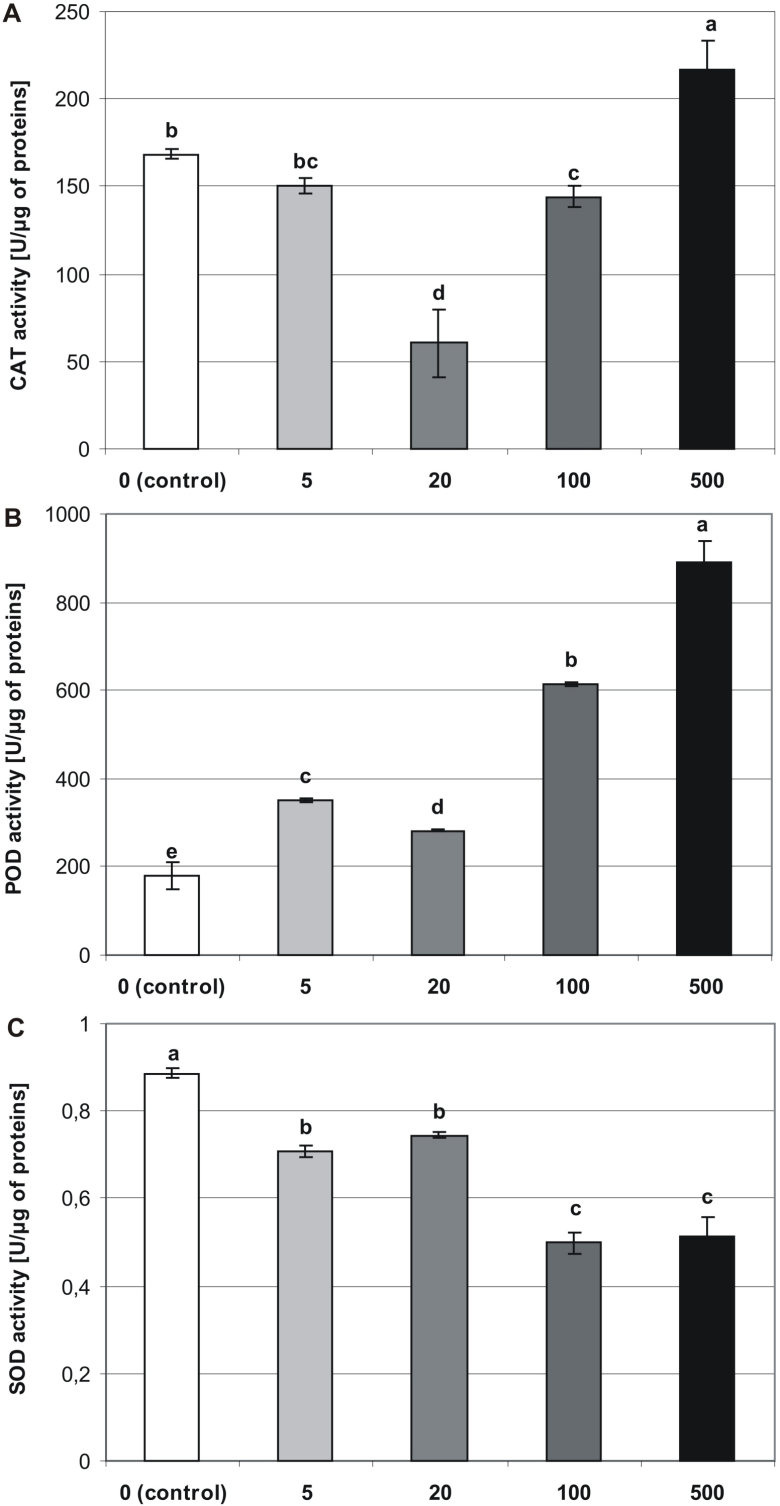
Effects of MEL on antioxidant enzymes activity in four-week-old plantlets. (A) CAT, (B) POD and (C) SOD. Melatonin was used at the germination stage at concentrations of 0, 5, 20, 100 and 500 µM, and the seeds were pre-incubated in darkness for 24 h. The results are the means of three replicates (*n* = 3) ±SE. Different letters indicate a significant difference at *p* < 0.05 according to ANOVA and Duncan’s test.

Melatonin applied at the germination stage, altered the activities of CAT, POD, and SOD in four-week-old stevia plantlets, and this effect depended on the concentration of MEL (*p* < 0.001, Table 1). The activity of CAT (Fig. 6A) was highest in plantlets obtained from seeds germinated on 500 µM MEL (216.65 U/µg of proteins). The remaining MEL concentrations decreased the activity of CAT in plantlets as compared with those of the control. The lowest level of CAT activity was observed in the case when 20 µM MEL was added at the germination stage (60.46 U/µg of proteins).

The analysis revealed that MEL contributed to an increase in the activity of POD (Fig. 6B). Consequently, the highest POD activity was observed in plantlets obtained from seeds germinated on 500 µM MEL (889.66 U/µg of proteins), and the lowest level of activity was detected in control plantlets (179.01 U/µg of proteins). Among the various concentrations of MEL tested, 20 µM had the greatest inhibitory effect on POD activity.

Superoxide dismutase activity significantly decreased in accordance with an increase in the MEL concentration (Fig. 6C). Consequently, the highest activity was observed in control plantlets (0.89 U/µg of proteins), and the lowest was found in plantlets obtained from seeds germinated on AG containing 100 and 500 µM MEL (0.5 and 0.51 U/µg of proteins, respectively).

## Discussion

Melatonin is a promising agent to improve stevia seed germination. In this study, the lowest MEL concentrations (5 and 20 µM) exerted a significantly positive effect on seed germination as compared with the highest concentrations (100 and 500 µM). Some previous studies indicated that the effect of MEL on seed germination depended on its concentration. Higher concentrations of MEL inhibited or had no influence on seed germination, whereas lower concentrations improved seed germination (Hernandez-Ruiz, Cano & Arnao, 2004; Chen et al., 2009; Wei et al., 2015). Posmyk et al. (2008) reported that concentrations of 1 and 10 µM of MEL appeared to have a beneficial effect on germination of red cabbage seeds under optimal conditions. On the other hand, another report pointed to MEL having no effects on germination of cucumber seeds, regardless of the germination conditions (Zhang et al., 2013).

Due to the sensitivity of MEL to light, in the present study, stevia seeds were pre-incubated in darkness*.* Pre-incubation periods of 24 h and 48 h were tested. As stevia is a positively photoblastic plant (Abdullateef & Osman, 2011; Kumar & Sharma, 2012; Simlat et al., 2016), germination without any pre-incubation was also performed. In all the pre-incubation groups, germination was performed in light conditions. In this study, MEL significantly increased stevia seed germination only after 24 h of pre-incubation. This GC was also higher compared to the GC of non-pre-incubated seeds. A longer pre-incubation period (48 h) was not associated with an increase in seed germination. In previous research on the effect of MEL on germination of red cabbage, cucumber, phacelia, or soybean seeds (Posmyk et al., 2008; Posmyk et al., 2009; Tiryaki & Keles, 2012; Wei et al., 2015), the seeds were primed in MEL solutions in darkness, and germination took place in darkness in accordance with the requirements of these species.

Previous research also demonstrated the regulatory role of MEL in plant growth and development in lupin and some monocotyledonous species (Hernandez-Ruiz, Cano & Arnao, 2004; (Hernandez-Ruiz, Cano & Arnao, 2005). In the current study, MEL had an effect on the aboveground parts of stevia plantlets: FW, stem length, and number of leaves. However, these effects depended on the MEL concentration. At a concentration of 500 µM, MEL inhibited not only germination but also subsequent growth of the aboveground parts of stevia seedlings. These findings are consistent with those of Posmyk et al. (2008), who showed that MEL at a concentration of 100 µM was toxic to the growth of red cabbage seedlings as compared with MEL at concentrations of 1 and 10 µM. In another study, Posmyk et al. (2009) confirmed that excessive MEL levels (500 µM) accelerated protein oxidation and caused problems with protein synthesis, possibly pointing to a pro-oxidative action of MEL at higher concentrations (Kładna, Aboul-Enien & Kruk, 2003). Bajwa, Shukla & Sherif (2014) also reported that the application of low concentrations (10–40 µM) of MEL increased the FW of Arabidopsis as compared with high concentrations (200–400 µM). Another study provided evidence that MEL promoted the vegetative growth and development of *Glycyrrhiza uralensis* (Afreen, Zobayed & Kozai, 2006). On the other hand, Shi et al. (2015a) and Shi et al. (2015c) found no significant difference between MEL-treated and non-treated plants. In the present study, a high concentration of MEL (500 µM) significantly influenced the root system and root number but inhibited root length. A previous study (Aguilera et al., 2015) reported that MEL significantly enhanced root development in lentil and kidney bean sprouts: radicles of lentils treated with 20 µM MEL were 1.4 times longer than those of lentils treated with H_2_O (control). In the same study, the radicals of kidney beans treated with MEL were 1.6 times longer than those of the control. As reported by Tan et al. (2012) in research carried out by Posmyk, corn seedlings obtained from MEL-treated seeds had a larger root system, and plants developed larger cobs than those of non-treated plants. The positive effect of MEL on the root system was also described in transgenic rice plants with high levels of endogenic MEL (Kang et al., 2010) and in *in vitro* cultures of sweet cherry (Sarropoulou, Therios & Dimassi-Theriou, 2012). However, the mechanisms by which MEL promotes root growth and development remain unknown. One possibility is that a high concentration of MEL might reduce the level of ROS in root cells. Sarropoulou, Therios & Dimassi-Theriou (2012) reported that low concentrations of MEL increased the number of roots and affected the root length and rooting percentage in commercial cherry rootstocks but inhibited root growth at higher concentrations. They attributed this finding to indole 3-acetic acid (IAA) synthesis. Their findings were consistent with those of Arnao & Hernandez-Ruiz (2006; 2007). In a recently published review, Arnao & Hernandez-Ruiz (2017; 2018) show that MEL behaves in a similar way to auxin. However, whether MEL induces auxin biosynthesis and whether it can be metabolized and converted to IAA remain unknown. As demonstrated previously (De Klerk, van der Krieken & De Jong, 1999), auxin is required only for root initiation, not root growth. These findings might explain the inhibition of root elongation by MEL at the highest concentration in the present study. Inhibition of root elongation in some monocots by MEL has also been reported (Hernandez-Ruiz, Cano & Arnao, 2005). In addition, as reported by Chen et al. (2009), the response of roots to MEL varied depending not only on the concentration but also the stage of growth and the genotype.

Chlorophyll is the major molecule responsible for photosynthesis. However, this molecule is fragile and easily injured by ROS, which are generated mainly during photosynthesis (Tan et al., 2012). In the present study, MEL affected the chlorophyll content, and these effects were dependent on the concentration. The levels of Chl *a* and Chl *b* were highest in stevia plantlets obtained from seeds germinated on 5 µM MEL. Similar results were observed for carotenoids. Higher concentrations of MEL also had a positive influence on the pigment content compared with that of the control, although the differences were significant only for Chl *a*. The role of MEL in protecting against chlorophyll degradation has been described previously (Arnao & Hernandez-Ruiz, 2009; Zhang et al., 2011; Wang et al., 2013a; Lazár et al., 2013), indicating that in addition to providing protection against ROS, MEL also affected chlorophylls and photosynthetic proteins. Arnao & Hernandez-Ruiz (2009) and Wang et al. (2013a) reported that MEL slowed the degradation of chlorophyll during senescence of barley and apple leaves, respectively. However, as reported by Wei et al. (2015), under normal conditions, the chlorophyll content of MEL-treated plants was similar to that of untreated plants.

Soluble sugars and phenolics are indicators of oxidative stress. In the present study, stevia plantlets obtained from seeds germinated on 500 µM MEL had the highest soluble sugar and phenolic contents. This finding may be explained by the occurrence of oxidative stress at higher concentrations of MEL. Soluble sugars might function as true ROS scavengers in plants, especially when present at higher concentrations. Phenolic compounds also play a vital role in protecting cellular membranes against the deleterious effects of ROS. According to Posmyk et al. (2009), MEL at high concentrations was associated with pro-oxidant activity in cucumber. Shi et al. (2015b) reported that pre-treatment of Arabidopsis plants with MEL (50 µM) significantly increased the accumulation of soluble sugars as well as the expression of stress-responsive genes. Szafrańska, Glińska & Janas (2012) suggested that MEL protected the roots of *Vigna radiate*, after re-warming chilled seedlings by increasing the synthesis of phenolic compounds. Dawood & El-Awadi (2015) also detected a higher content of phenolics in MEL-treated *Vicia faba* seedlings.

Melatonin may also act as an antioxidant by increasing the activities of ROS-scavenging enzymes. As observed in the present study, at the highest concentration (500 µM), MEL significantly increased the activity of CAT and POD in stevia plantlets, whereas it reduced the activity of SOD. This finding suggested that a high concentration of MEL led to increased stress in stevia plantlets. A concentration of 500 µM of MEL was unfavorable for both seed germination and further growth of seedlings and plantlet development. Low CAT and POD activities observed in plantlets obtained from seeds germinated on AG with 20 µM MEL correlated with improved seed germination, high FW increments, and increased numbers of leaves. As reported earlier, MEL increased the activities of several antioxidant enzymes, including CAT, POD, and SOD, and endogenous MEL appeared to play a role in the first line of defense against oxidative stress. Jeng & Sung (1994) suggested that CAT, POD, and SOD enzymes may reverse lipid peroxidation, mediated by free radicals. Zhang et al. (2014) reported that under stressful conditions MEL affected antioxidant enzyme activities by increasing the expression of their corresponding genes. However, Shi et al. (2015a) and Shi et al. (2015c) found no significant effects of MEL on the activities of antioxidant enzymes under control conditions. In contrast, Wang et al. (2016) indicated that pre-treatment of cucumber seedlings with MEL enhanced the activity of antioxidant enzymes CAT, POD, SOD, and APX (ascorbate peroxidase; EC1.11.1.11).

## Conclusions

The positive effect of seed priming in MEL solutions on seed germination and plant quality, especially under stressful conditions, is well documented. The results of this research suggested that MEL added to the germination substrate at lower concentrations had a stimulating effect on germination compared to the higher concentrations, which inhibited seed germination and led to the increased stress in the plantlets of stevia. The MEL concentration of 20 µM was optimal for seed germination as well as for morphological parameters of plantlets (higher weight increments, increased number of leaves). This concentration also resulted in relatively low levels of soluble sugars, phenolic compounds as well as low activity of CAT and POD.

##  Supplemental Information

10.7717/peerj.5009/supp-1 Data S1 Experimental data

## Abbreviations

AG: agar gel
CAT: catalase
dH_2_O: distilled water
EDTA: ethylenediaminetetraacetic acid
FW: fresh weight
GC: germination capacity
GE: germination energy
H_2_O_2_: hydrogen peroxide
MEL: melatonin
MS: Murashige and Skoog medium
POD: peroxidase
ROS: reactive oxygen species
SE: standard error
SOD: superoxide dismutase.

## Additional Information and Declarations

### Data Availability

The following information was supplied regarding data availability: The raw data are provided in Data S1.
